# Unmasking a Silent Threat: Office-Based Diagnosis of an Ascending Aortic Dissection With Transthoracic Echocardiography

**DOI:** 10.7759/cureus.60610

**Published:** 2024-05-19

**Authors:** Marc T Zughaib, Phanindra Antharam, Andrew D Assaf, Marcel E Zughaib

**Affiliations:** 1 Cardiology, Ascension Providence Hospital, Southfield, USA

**Keywords:** hypertension, aortic dissection, cardiology, transthoracic echocardiogram, type a aortic dissection

## Abstract

Type A aortic dissection (TAAD) is a potentially life-threatening diagnosis that can present with elusive symptomatology. A high degree of clinical suspicion is necessary for prompt diagnosis and management. We describe a case of a transthoracic echo (TTE) in a non-suspicious clinic patient diagnosed with TAAD. A 66-year-old Caucasian male presented for a routine clinic visit with one episode of acute severe chest pain. An echocardiogram was ordered for further workup of hypertension and chest discomfort. The echocardiogram demonstrated an ejection fraction of 60% without significant valvular abnormalities. There was suspicion of aortic pathology, which required multiple attending to review the images. The final interpretation was TAAD with a thrombus present in the false lumen. The patient then presented to the Emergency Department. A computed tomographic angiography was performed, which subsequently confirmed the TAAD. The patient was admitted to the cardiovascular ICU and ultimately underwent a successful repair of the dissection. The patient had an unremarkable post-operative course and was ultimately discharged home. Our case demonstrated a diagnosis of TAAD by office-based TTE as the original imaging modality. While this was unconventional, a TAAD should remain on the differential diagnosis when being ordered for the patient's with uncontrolled hypertension with chest pain as a presenting symptom.

## Introduction

The Stanford classification of aortic dissection was described in 1970, which described a type A aortic dissection (TAAD) involving the ascending aorta and a type B aortic dissection (TBAD) involving the descending aorta [1]. Type A acute aortic dissection is a life-threatening emergency that requires prompt surgical intervention and carries high mortality and morbidity [2]. As symptoms mimic other common disorders such as myocardial ischemia, a missed or delayed diagnosis is not uncommon.

Aortic dissection has an incidence of 4.4 per 100,000 person-years and sometimes is diagnosed postmortem [3-5]. Risk factors include hypertension, atherosclerosis, iatrogenic after cardiac catheterization, prior cardiac/aortic surgery, aortic aneurysm, connective tissue disorders, and bicuspid aortic valve [6,7]. A younger age at presentation is more likely secondary to connective tissue disorder or familial dissections [8].

Since the 1970s, diagnostic tools and management of acute type A aortic dissection have undergone substantial evolution with computed tomography (CT) as the gold standard [1]. We present a case of an acute TAAD, identified incidentally during a routine outpatient echocardiographic evaluation, and discuss the relevance of transthoracic echocardiography (TTE) in its diagnosis.

## Case presentation

A 66-year-old Caucasian male, with a past medical history of hypertension, binge alcohol use disorder, and coronary artery disease with prior percutaneous coronary intervention and stent placement for in-stent restenosis of the right coronary artery seven months prior, was seen in the outpatient cardiology clinic for an episode of severe chest pain. The patient reported experiencing chest pain two weeks prior to presentation, which occurred at rest with radiation to the jaw. His pain lasted about 30 seconds and subsequently resolved with isosorbide dinitrate. His chest symptoms did not recur after the initial episode. However, the patient had been limiting his overall activity. He additionally experienced palpitations and night sweats after the initial episode. The review of systems was otherwise negative. There was no significant family history, surgical history, or allergies. Medications included aspirin of 81 mg, atorvastatin of 80 mg daily, isosorbide dinitrate of 30 mg daily, and clonazepam as needed. He was not receiving beta-blockers due to prior episodes of bradycardia. Vitals at presentation in the office were blood pressure of 160/92 mmHg in the left arm and 162/98 mmHg in the right arm and a heart rate of 76/min. Overall, the physical examination was unremarkable.

**Figure 1:**
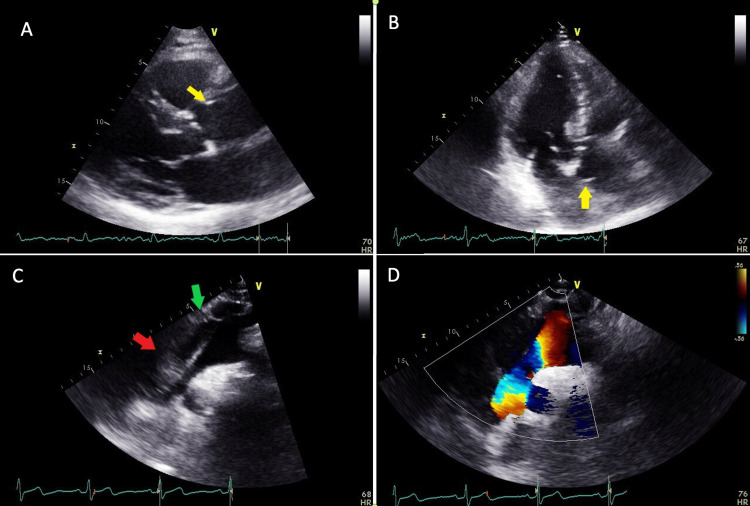
Echocardiogram images of the ascending type A dissection. Panel A: Parasternal long-axis view showing the dissection flap (yellow arrow) and dilated ascending aorta. Panel B: Apical three-chamber view showing the dissection flap (yellow arrow). Panel C: Suprasternal view showing the false lumen of the dissection (green arrow). Panel D: Suprasternal view showing no flow on color Doppler in the false lumen.

An ECG performed on admission demonstrated normal sinus rhythm without ischemic changes. The patient subsequently underwent a transthoracic echocardiogram (TTE) for further workup of the possible acute coronary syndrome. This TTE demonstrated an ejection fraction (EF) of 55-60%, apical akinesis consistent with previous myocardial infarction, no significant valvular abnormalities, and a possible aortic dissection on the supra sternal view (Figure 1). The aortic root was reported as measuring 4.9 cm, with the ascending aorta (AA) measuring 5.6 cm. Several attending cardiologists were asked to interpret the images due to the unlikely nature of a patient presenting to the office with a stable TAAD. However, the final interpretation was determined to be a TAAD with a thrombus present in the false lumen. Due to the findings above, he was advised to present to the Emergency Department and started on an esmolol drip immediately.

**Figure 2:**
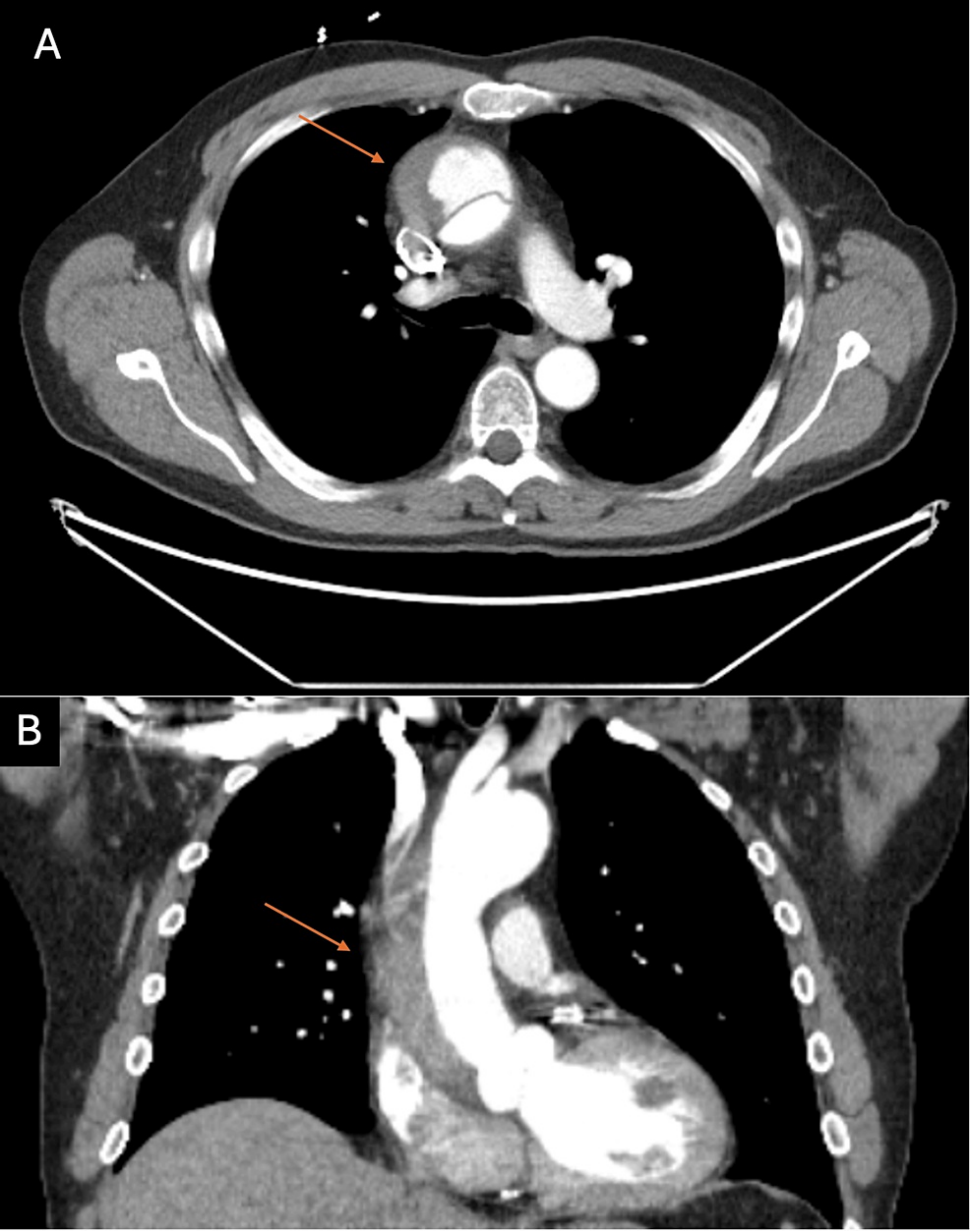
CT angiogram with type A aortic dissection with aneurysmal dilation of 5.3 x 5.4 cm at maximal diameter. False lumen on the patient's right (orange arrows) and true lumen opacified with contrast. Panel A: Transverse axis view of TAAD extending from the aortic root up to the distal aortic arch with intimal tears noted within the arch. Panel B: Coronal axis view of TAAD.

Lab studies demonstrated a normal troponin, white blood cell count, hemoglobin, platelet level, creatinine, and electrolytes. The electrocardiogram demonstrated normal sinus rhythm with a rate of 70, normal axis, and nonspecific ST-T wave changes. Computed tomographic angiography (CTA) of the chest-abdomen-pelvis with contrast revealed a fusiform aneurysmal dilatation of the AA and TAAD extending from the aortic root up to the distal aortic arch with intimal tears noted within the arch (Figure 2). There was no opacification of false lumen thought to be secondary to intramural hematoma/partial thrombosis/slow flow. The AA was measured at 5.3 x 5.4 cm at maximal diameter. There was no evidence of pericardial effusion, and a calcified plaque was noted within the iliac arteries with no evidence of high-grade luminal stenosis.

The patient was initially managed medically with an esmolol drip in the cardiovascular ICU. Cardiothoracic surgery was consulted, and emergent surgical repair with replacement of ascending aorta with a hemi-arch graft was done two days after the initial TTE. Intra-procedure, the ascending aorta was noted to be recently dissected consistent with the CTA findings. A large amount of clot was removed from the false lumen. No dissection was noted down the left coronary cusp. The dissection flap spiraled anteriorly ending and artificially separating the left subclavian and left carotid arteries. No intraoperative complications occurred. Intraoperative transesophageal echocardiography showed an EF of 45%, no significant valvular abnormalities, and no significant change postoperatively. He was discharged in a stable condition on post-operative day seven.

## Discussion

The most common symptom and characteristic clinical presentation of acute aortic dissection is sharp tearing chest pain in the anterior chest wall most likely being TAAD vs the posterior chest wall being type B aortic dissection (TBAD). Although characteristic, the pain may mimic other conditions, especially acute myocardial ischemia, and the diagnosis may be missed or delayed, resulting in increased morbidity and mortality [2].

The TTE for our patient was ordered as part of the evaluation of ischemic heart disease, as well as uncontrolled hypertension. Given the clinical presentation and outpatient clinical setting with a very low pretest probability, this potentially highly fatal condition could have been missed. The diagnostic difficulty within this patient's presentation was related to the low likelihood of a stable TAAD presenting to the office several weeks after symptom onset. The diagnosis required several separate reviews by attending cardiologists.

A high degree of clinical suspicion as the diagnosis of acute TAAD early at presentation is of paramount importance given the high rate of mortality (up to 44%) [8]. High-risk clinical features include abrupt onset of thoracic or abdominal pain with sharp tearing or ripping character, variation in pulse, or complete absence or difference of systolic blood pressure > 20 mmHg between the two arms, which should prompt further evaluation [9]. Historically, sensitivity in the diagnosis of TAAD with a TTE was 78-90% and a specificity ranging from 87 to 96% [10,11]. In contrast, TEE demonstrates a sensitivity and specificity of 98% and 96%, respectively [12,13]. The accuracy of CT and MRI in the diagnosis of aortic dissection is high, with sensitivity and specificity ranging around 98-100% [14]. Ultimately, our patient was able to have the correct clinical diagnosis, which led to the appropriate and timely surgical management.

## Conclusions

In this clinical case, our patient had severe chest pain symptoms highly suspicious for acute coronary syndrome with other etiologies less likely. However, he did not seek emergent/urgent evaluation as his chest pain resolved. An aortic dissection was an unexpected finding as the patient was hemodynamically stable and asymptomatic at the time of evaluation in the outpatient clinic. The TTE was critical in the early diagnosis of this potentially fatal dissection. This case not only highlights an atypical presentation of a chronic, stable TAAD, but it also stresses the continued importance of high levels of suspicion for life-threatening emergencies.
